# A randomized clinical trial to test efficacy of chamomile and saffron for neuroprotective and anti-inflammatory responses in depressive patients

**DOI:** 10.1016/j.heliyon.2022.e10774

**Published:** 2022-09-30

**Authors:** Saara Ahmad, Arfa Azhar, Prashant Tikmani, Hamna Rafique, Asra Khan, Hanif Mesiya, Humera Saeed

**Affiliations:** a Department of Biological and Biomedical Sciences, Aga Khan University, Karachi, Pakistan; b Department of Psychiatry, Aga Khan University, Karachi, Pakistan

**Keywords:** Brain derived neurotrophic factors (BDNF), Chamomile, C-reactive protein (CRP), Depression, Patient health questionnaires 9 (PHQ-9), Saffron, Tryptophan (TRP)

## Abstract

Depression is one of the common psychiatric problems in growing world population caused by long-term stressful events that may trigger the down regulation of neurogenesis. The pathogenesis of depression initially relies on serotonin deficiency which is associated with depressive feelings. Tryptophan (TRP) depletion participate crucial role in inducing depressive symptoms. Long-term reduction of 5-HT may disseminate to high sensitivity of MDD and alters the level of BDNF. Some studies have also revealed the strong association between excessive neuroinflammation and BDNF levels, due the release of pro-inflammatory cytokines. The treatment approach through FDA approved medicine has their own merits and drawbacks. Therefore, herbal alternatives have recently garnered attention for their effectiveness against depression. However, evidence-based synergic effects of antidepressant with different herbal agents are limited. The purpose of this study was to assess the synergistic effects of two well-known herbs, chamomile and saffron, as an adjuvant therapy in patients with mild to moderate depression. The present study was study randomized, open, blinded trial and comprised of 120 participants randomly allocated to control (n = 60) and test (n = 60). After consent, the patient health questionnaire- 9 (PHQ-9) was filled to obtain depression scores. The test participants were received herbal tea sachets twice a day for one month (20 mg Chamomile and 1 mg Saffron/sachet) along with routine medicines, while control participants were received only allopathic medications. Blood samples were taken before and after the treatment. The depressive symptoms improved significantly with both treatments. The effect of herbs enhanced the efficacy of medications and significantly improved PHQ-9 scale and BDNF while reduced the inflammatory markers (CRP) and TRP level in plasma thereby increased the availability of TRP in brain. It has been concluded that the herbal adjuvant therapy produced long term improvement against depression and enhanced the efficacy of allopathic treatment.

## 1 Introduction

Depression is one of the typical disorders effecting 5% of global population, represented in several clinical forms. Long-term stressful events may cause the triggering effects by down regulation of neurogenesis and initiation of depression. Globally, more than 20% of people affected by mental illness and depression is one of the most common incapacitating disorders which affect the quality of life. It may occur at any age and mainly marked by persistent sadness or anhedonia, disrupting psychosocial functions and sleep/appetite pattern, also known as major depressive disorder (MDD). MDD is listed as 3rd largest cause of disease burden and supposed to rank 1st by 2030 (Zhu et al., 2020). Because of its diverse clinical presentation and etiology, understanding the pathophysiology of depression is a challenging task. The symptoms of depression are more likely to appear as disruptive behavior and personality disorders. The pathogenesis of depression initially relies on serotonin hypothesis. Serotonin is a neurotransmitter that regulates a variety of physiological functions in the brain, including behavior, learning, mood, and appetite. The deficiency of serotonin (5-hydroxytryptamine, 5-HT) in brain is associated with pessimistic thoughts, self-accusation, fear, hostility, loneliness, and depressive feelings. It suppresses the behavioral response of pain and aversive stimuli and enhances anti-aggressive response. Many studies reported the serotonergic decline in serum samples of depression patients as compared to healthy subjects (Bot et al., 2015; Coppen, 1967; Hogenelst et al., 2016; Phillips, 2017). Other findings are indicating that the tryptophan (TRP) depletion may also induce relapse. TRP is essential amino acid found from protein-based diet, it uses a particular transporter to pass the blood-brain barrier in competition with other large neutral amino acids (LNNAs) for access to the brain. TRP is hydroxylated by the rate-limiting enzyme tryptophan hydroxylase (TPH) and then decarboxylated by aromatic acid decarboxylase in serotonin-producing cells. TPH is the first enzyme in the brain serotonin production pathway, and it is only half-saturated by typical physiological TRP concentrations, making it a rate-limiting step (Zhang et al., 2006) therefore, the increase level of TRP increases the production of 5-HT up to two -folds. There are growing evidence showing that the depletion of TRP increases the depressive symptoms (Ruddick et al., 2006). Some evidence also suggested the impaired function of cytokines in the induction of major depressive disorder (Petralia et al., 2020a, 2020b). These are the special proteins released from immune systems and exert their function against the development of tumors and autoimmune diseases. Several studies have shown that the depressive patients may also report with abnormal immunological parameters including tumor necrosis factor (TNF) and interleukins (Ruiz et al., 2022).

Many studies highlighted the role of hypothalamus-pituitary-adrenal (HPA) axis, which is the major endocrine stress system, is frequently dysregulated in MDD patients, the upregulation of the hypothalamus-pituitary-adrenal (HPA) axis (Mikulska et al., 2021). The circulating cortisol due to excessive stress may exaggerate the neurotoxic effects on hippocampus and blunt the production of serotonin by downregulating the neuronal firing of 5-HT neuron projecting into Prefrontal cortex (PFC) from raphe nuclei in brain stem (Mahar et al., 2014). Long-term reduction of 5-HT may disseminate to high sensitivity of MDD and alters the level of neurotropic factors which are responsible for the neuronal growth and plasticity particularly BDNF. Brain derived neurotrophic factor (BDNF), belongs to the family of neurotrophins growth factor which is widely distributed in the central nervous system. It helps in survival and growth of nervous system by the activation of 3 signaling pathways i.e., Mitogen-activated protein kinase (MAPK), phosphoinositide 3-kinase and phospholipase C-which are bound to its receptor tropomyosin receptor kinase B. (TrkB) (Saarelainen et al., 2003). BDNF is synthesized as pro-BDNF mostly in hypothalamus and hippocampus, which is then cleaved to form mature BDNF and pro peptide which exhibit numerous biological activities. During depolarization, the mature neurons and glial cells cause the synthesis and release of BDNF along with other neurotrophins (Rosso et al., 2017). It is been suggested that uneven levels of pro-BDNF and mature BDNF cause the neuronal death and behavioral disorders (Yang et al., 2020). Numerous studies have found an association between neurotrophic growth factors, particularly BDNF, and depression. BDNF regulates activity-dependent changes in synapse structure and function as well as neurogenesis. Disturbance or a reduction in BDNF causes neuronal plasticity to malfunction, as well as a reduction in excitatory neurons and glutamate, which can lead to depression (Arumugam et al., 2017). A few studies, on the other hand, found no significant difference in BDNF blood levels between MDD patients and controls (Haapakoski et al., 2015). Some studies have also revealed the strong association between excessive neuroinflammation and BDNF levels, due the release of pro-inflammatory cytokines. C-reactive protein (CRP) is one of the members of acute phase protein which is widely used for the diagnosis of depression in many previous studies. A high-sensitivity CRP assay is well-validated and widely available. CRP synthesis is induced by the proinflammatory cytokines in liver as a result of infection, inflammation, or tissue damage. CRP was found to be moderately elevated “on average” in patients with MDD in a meta-analysis of 20 case–control studies (Glaser et al., 2003). The size of the effect, however, varied significantly between studies, this could be due to clinical heterogeneity, as severe depression has a higher CRP than mild/moderate depression, as well as differences in study methodology (Florea et al., 2015).

There are several lines of medications available for the treatment of MDD, they are categorized in different classes including tricyclic antidepressant (TCA), monoamine oxidase inhibitor (MAOIs), selective serotonin reuptake inhibitors (SSRIs) and selective norepinephrine reuptake inhibitors (SNRIs) (Montgomery et al., 2014). The appropriate treatment and prescribing pattern of antidepressants may vary based on the requirement of the patient. However, the replacement of TCA and MAOIs drugs by SSRIs. SSRIs and SNRIs are reported as appropriate option with better tolerance and broader indications for the treatment of MDD and anxiety (Al Shweiki et al., 2019). Despite this, most depressed people continue to experience persistent symptoms. Antidepressants, upon optimal use, can help to alleviate distress and depressive symptoms. Due to the difficulty of treating a disease with a single target, a multiple targeting system method has been reported to have reliable results. Sequential treatment of SSRI and another antidepressant is also widely recommended for the treatment of MDD and has been shown to be more effective than monotherapy (Wang et al., 2018). However, the chronic use of SSRIs and SNRIs are also associated with a range of side effects including drowsiness, fatigue, nausea, weight loss and cardiovascular problems (Wong et al., 2017). Thus, the treatment approach through FDA approved medicine has their own merits and drawbacks. Therefore, herbal alternatives have recently garnered attention for their effectiveness against depression. However, evidence-based synergic effects of antidepressant with different herbal agents are limited (Ahmad et al., 2020).

Saffron (*Crocus sativus* L.) is a promising candidate, as the dried stigma used for several years for the treatment of various ailments (Roshanravan et al., 2020). The stigma contains large number of bioactive agents named crocin, crocetin and carotenoids including picrocrocin and safranal which are responsible for bitter taste and aroma respectively. The compounds of saffron immensely displayed antioxidant, anti-inflammatory and antidepressant properties. A study defined the effects of saffron doses (30 mg/kg) on depression patients and revealed that the antidepressant effects of saffron are strongly as effective as conventional medications (antidepressants), suggesting the potential modulation of HPA axis (Ghadrdoost et al., 2011). Based on literature, it is supposed that the saffron shares the pharmacological effects with antidepressants and restore the levels of 5-HT. The link between changes in neuroplasticity and depression has recently gained a lot of attention and it has been reported in many studies that BDNF level are strongly affected in depression. In a study, rats exposed to the forced swimming test, the neuroprotective and antidepressant effects of saffron and crocin (a component of saffron) were investigated, and results revealed that crocin dose-dependently increased levels of BDNF in the hippocampus and exert antidepressant effects (Shahmansouri et al., 2014; Vahdati Hassani et al., 2014). However, the promising anti-inflammatory effects, the extract of saffron is reported to reduce inflammatory markers via inhibition the nuclear factor erythroid 2–related factor 2 (NF-κB) (Zhang et al., 2015). *Matricaria chamomilla* L. is another world’s best known and well-studied medicinal plant (Brendler, 2006). Chamomile tea is made from dried flower heads which have traditionally been used for numerous therapeutic purposes. There are many active components in flower of chamomile including apigenin, quercetin, patuletin, luteolin, and their glucosides (McKay and Blumberg, 2006). Chamomile is best known for its anti-inflammatory, antioxidant, anti-cancer, neuroprotective, anti-allergic, and anti-microbial properties (Chandrashekhar et al., 2011; Patel et al., 2007; Ranpariya et al., 2011; Sebai et al., 2014; Silva et al., 2012; Zemestani et al., 2016). The effect of chamomile in depression is uncertain; however, it may be independent of its anxiolytic function (Amsterdam et al., 2009). Several studies suggest that active component present in chamomile may have an antidepressant effect through controlling catecholamines, serotonin (5-HT), and -amino butyric acid (GABA) neurotransmission (Yi et al., 2008). Moreover, chamomile appears to modulate hypothalamic-pituitary- adrenocortical (HPA) axis activity (Reis et al., 2006). Additionally, apigenin which is one of the major components of chamomile was reported to enhance norepinephrine activity and reduce monoamine oxidase (MAO) activity in an isolated rat atria model (Lorenzo et al., 1996). Therefore, the aim of the current study is to investigate the potential effects of combination of saffron and chamomile on depression through PHQ 9 scale and related parameters such as tryptophan, BDNF, CRP.

## 2 Materials and methods

This was a prospective, randomized, blinded end-point trial primarily to evaluate the efficacy of herbs in depressed patients. The study was approved by the Aga Khan University’s ethical review committee under the number 5011-BBS-ERC-17 and registered with ClinicalTrials.gov. Identifier: NCT04935671.

### 2.1 Collection of medicinal plants and preparation of herbal tea sachets

Dried floral parts of Chamomile (Baboona) and stamens of Saffron (Zafaran) were purchased from a local supermarket in Karachi, Pakistan and a sample was assigned the herbarium-voucher number MC- FL-08-18-05 for chamomile and CS-ST-08-18-05 for saffron and will be preserved at The Aga Khan University’s Natural Products Research Division, Department of Biological and Biomedical Sciences, Karachi. Tea bags containing 20 mg of chamomile and 1 mg of saffron were prepared. These were stapled in a sachet and given to patients who agreed to participate in the study.

### 2.2 Sample size

**Figure 1:**
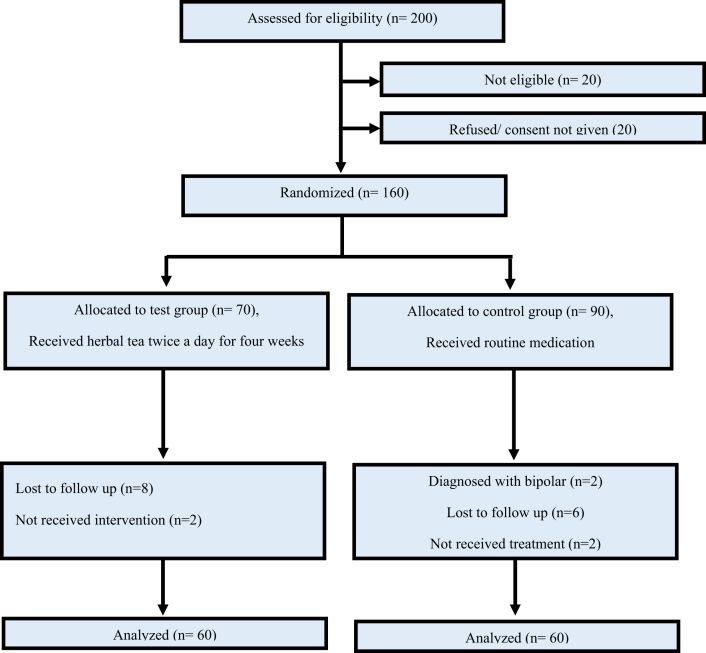
Flow chart of protocol.

The OpenEpi calculator, version 3, was used to compute the sample size in accordance with precision and efficiency concerns, and a sample size of 120 participants was selected (Figure 1).

### 2.3 Participants

Subjects were included if they met inclusion criteria: i) mild to moderate depression ii) with or without diabetes, hypertension and dyslipidemia iii) free from any psychiatric condition other than depression Subjects were excluded if they had i) substance dependence ii) terminal ailments/conditions like cancer, and morbid depression requiring hospitalization iii) for females if they were pregnant.

The total 120 patients were included in the study participated from Outpatient department of Psychiatric clinic of Aga Khan university Hospital. The trial was initiated from July 2020 and terminated by December 2020. The data collection ended in January 2021. The demographic details of patients are described in Table 1, collected from medical records provided by available nursing staff.

**Table 1:** Demographic details of patients.

Characteristics	Test n = 60	Control n = 60
Age (Mean ± SD)	65 (4.5)	67 (5.2)
Sex (M/F)	27/33	24/36
Marital Status		
Married	45	38
Separated/Divorced	4	10
Single	11	12
Education		
High Education	5	8
Graduate	33	28
Matric	22	24

### 2.4 Intervention

The individuals were assigned to one of two groups: control (n = 60) or test (n = 60) according to the randomization list provided by clinical trial unit (CTU) of Aga Khan University Karachi, responsible to increase the awareness about clinical trial and empower research team by interdisciplinary collaboration. All eligible individuals were assessed by a consultant psychiatrist at the Aga Khan University Hospital’s outpatient psychiatric clinic in Karachi, Pakistan. The patient health questionnaire-9 (PHQ-9) was given to all patients, and data was gathered by a blinded researcher who was unaware of the treatment allocation. The outcome variables were depression scores. Patients were randomly assigned to test or control groups after providing informed written consent. For one month, the test participants were given herbal tea by one of the unblinded Research Associate and directed to take as a decoction twice a day. The doses were chosen based on previous research suggesting that the herbs increase positive mood and help alleviate depression at various doses, with 30–100 mg of chamomile being effective for the management of depression in humans, and up to 30 mg of saffron producing effects like fluoxetine in depressed patients (Keefe et al., 2016). To avoid any untoward adverse effect of coadministration as an adjuvant therapy, a minimal therapeutic dose of saffron and chamomile of 20 mg and 1 mg respectively in a tea bag was given twice a day after meals for one month period. Blood samples were collected at Karachi’s Aga Khan Laboratory, and the PHQ-9 questionnaire was completed twice, once at the start of the trial and again at the end of the one-month period. Patients were not subjected to any dietary or lifestyle changes.

### 2.5 Outcome variables

**Primary outcomes:** The direct assessment of each patient was done by PHQ-9 questionnaires at baseline and post treatment period to compare the response of patient before and after treatment period by a blinded researcher. The mean scores were calculated to present the graphical data of both test and control group participant.

**Secondary outcomes:** Evaluation of efficacy of herbal tea was done by analyzing blood samples. Different parameters were used to observe the effects including Tryptophan, BDNF, CRP. The mean values were calculated to present the graphical diagram.

### 2.6 Biochemical analysis

#### 2.6.1 Estimation of tryptophan (TRP) level

Serum TRP was quantified using an ELISA method (Cloud Clone Corp. Tryptophan competitive ELISA kit, CED720Ge, USA) following the instructions of the manufacturer. The detectable range of the kit 1.23–100 ug/ml. The pre-coated with a tryptophan-specific antibody microplate were used as provided in the kit. With the pre-coated antibody specific to tryptophan, a competitive inhibitory process is initiated between biotin labeled tryptophan and unlabeled tryptophan (Standards or samples). The unbound conjugate is rinsed away after incubation. Each microplate well is then treated with avidin coupled to Horseradish Peroxidase (HRP). The amount of bound HRP conjugate in the sample is inversely related to the concentration of tryptophan. The intensity of the color formed after the addition of the substrate solution is inversely proportional to the concentration of tryptophan in the sample. The plate was read at 460 nm.

#### 2.6.2 Estimation of brain derived neurotropic factor (BDNF) level

Serum BDNF was quantified using an ELISA method (Cloud Clone Corp. BDNF Sandwich ELISA kit, SEA011Mi, USA) following the instructions of the manufacturer. The detectable range of the kit is 31.2-2000 pg/ml. The pre-coated with a BDNF-specific antibody microplate were used as provided in the kit. Then, using a biotin-conjugated antibody specific to BDNF, the standards or samples are applied to the relevant microplate wells. Following that, each microplate well is incubated with avidin coupled to horse-radish peroxidase (HRP). Only the wells containing BDNF, biotin-conjugated antibody, and enzyme-conjugated avidin will change color after the addition of 3,3′,5,5′-Tetramethylbenzidine (TMB) substrate solution. The enzyme-substrate reaction is stopped by adding sulphuric acid solution, and the color change was detected spectrophotometrically at 450 nm. The concentration of BDNF in the samples is then calculated by comparing the optical density of the samples to the standard curve.

#### 2.6.3 Estimation of C-reactive protein (CRP) level

Serum CRP was quantified using an ELISA direct sandwich method (Sigma Aldrich ELISA kit, SE120041, USA) following the instructions of the manufacturer. The detectable range of the kit 0.2–10 mg/L. The samples and anti-CRP-HRP conjugate were added in wells coated with CRP monoclonal antibody. CRP in the patient’s serum binds to the anti-CRP monoclonal antibody on the well, and the second anti-CRP antibody attaches to CRP. Wash buffer removes unbound protein and HRP conjugate. The intensity of color after the addition of the substrate is proportional to the concentration of CRP in the samples. A standard curve is created that relates color intensity to CRP concentration. The plate was detected at 450 nm.

#### 2.6.4 Statistical analysis

The data was analyzed using a one-way ANOVA. The PHQ-9, as well as TRP, BDNF, and CRP, were the key dependent measures. SPSS-25 software was used to conduct statistical analysis. The Bonferroni test was used to make pair-wise comparisons, and an alpha value of p ˂ 0.05 was considered significant.

## 3 Results

### 3.1 Effects on tryptophan levels

**Figure 2:**
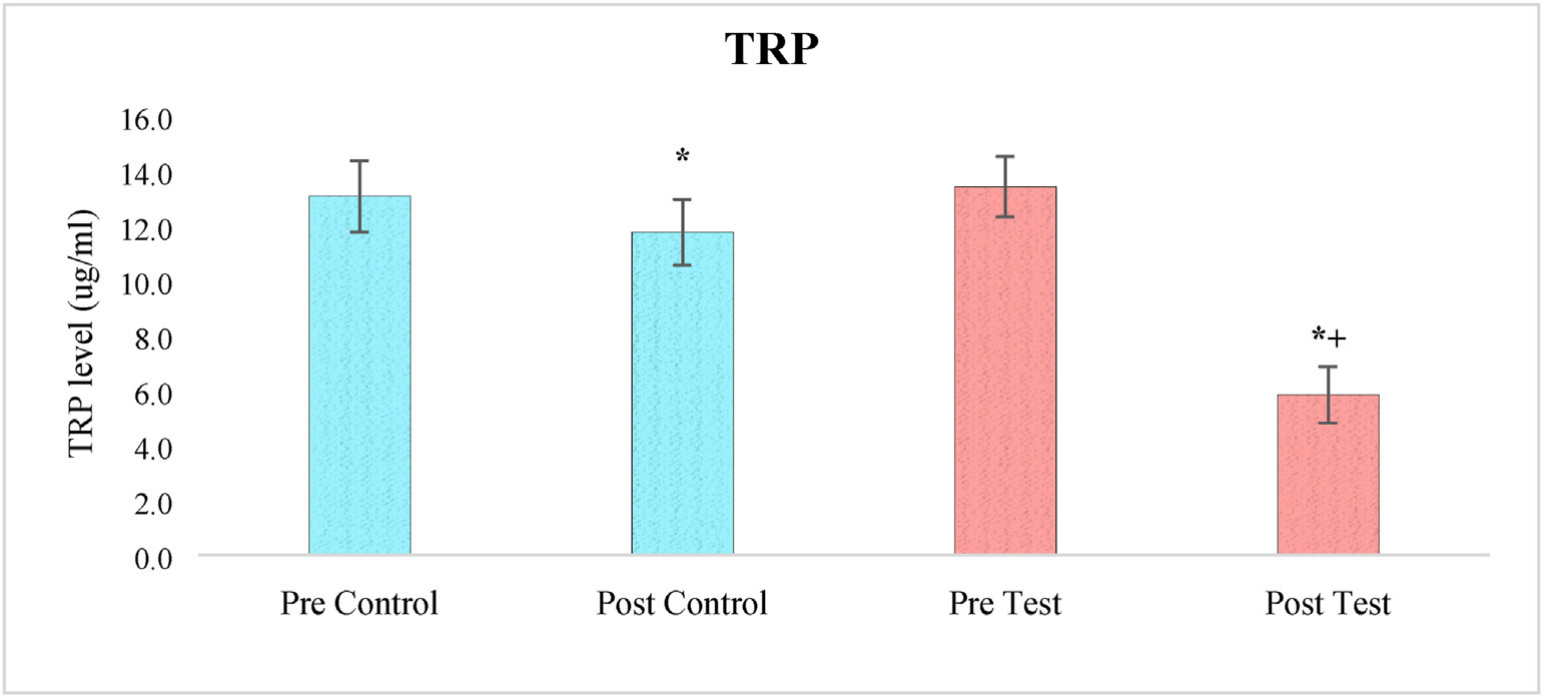
The effect of herbal tea on TRP levels. Values are presented as mean ± SD. Significant differences by Bonferroni test. ∗p < 0.05 as compared to respective pre-test group, +p < 0.05 as compared to post control group.

A one-way ANOVA analysis of tryptophan levels revealed a significant effect of treatment (F_3,236_ = 221.643, p < 0.05). At baseline, when the clinical trial began, pair-wise comparisons using the Bonferroni test revealed a significant difference in tryptophan levels between the two groups (test and control). When comparing before and after therapy, the amount of tryptophan in the test group was significantly lower (p < 0.05). The benefits of herbal medication in addition to allopathic treatment on lowering blood tryptophan levels in test individuals were very significant (p < 0.05) when compared to control participants who received one-month allopathic treatment only (Figure 2).

### 3.2 Effects on BDNF level

**Figure 3:**
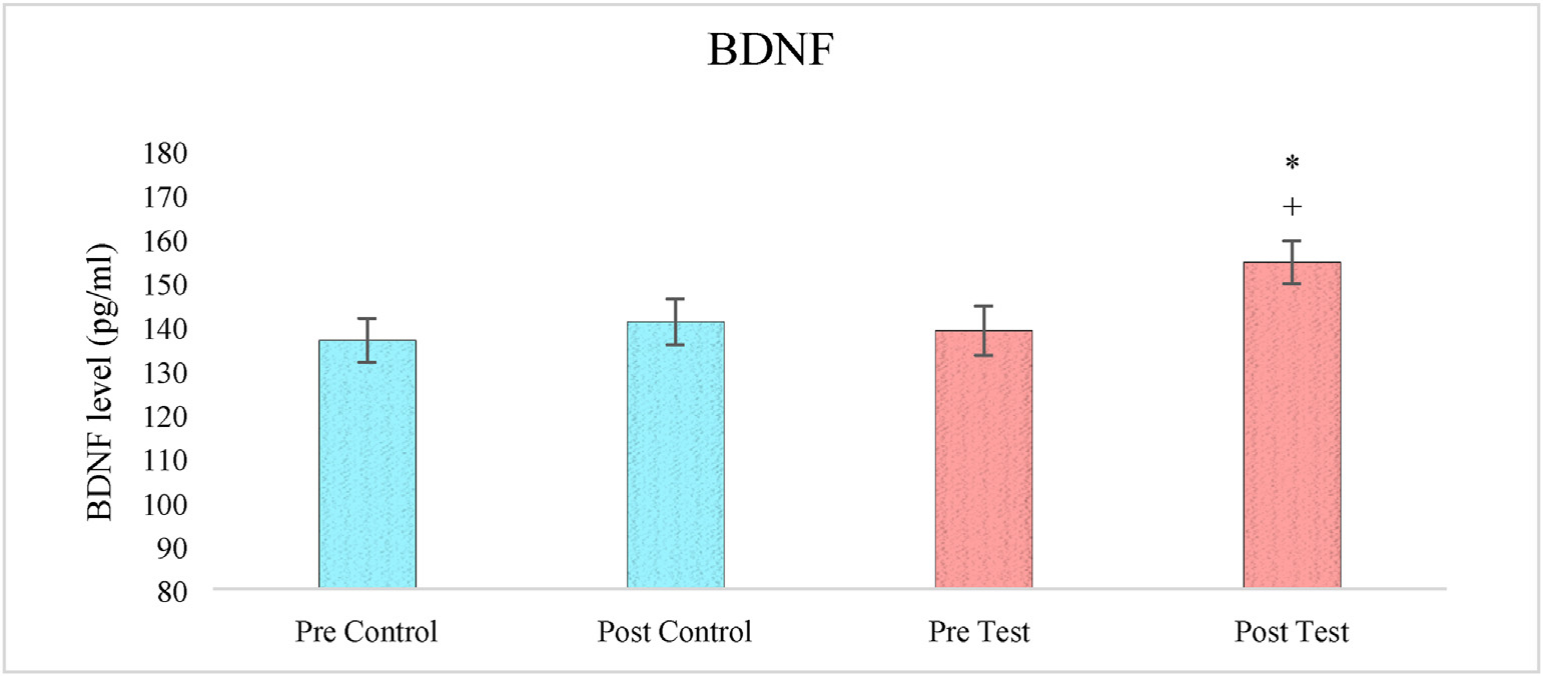
The effect of herbal tea on BDNF levels. Values are presented as mean ± SD. Significant differences by Bonferroni test. ∗p < 0.05 as compared to respective pre-test group, +p < 0.05 as compared to post control group.

A one-way ANOVA analysis of BDNF levels revealed a significant effect of treatment (F_3,236_ = 13.844, p < 0.05). Pair-wise comparisons using the Bonferroni test revealed a significantly elevated level of BDNF in test group patients after four weeks of adjuvant therapy (p < 0.05) when compared to pre-test levels. When compared to control participants, the effect of herbal medication in addition to allopathic treatment on elevating blood BDNF levels in test subjects were quite significant (p < 0.05) (Figure 3).

### 3.3 Effects on CRP levels

**Figure 4:**
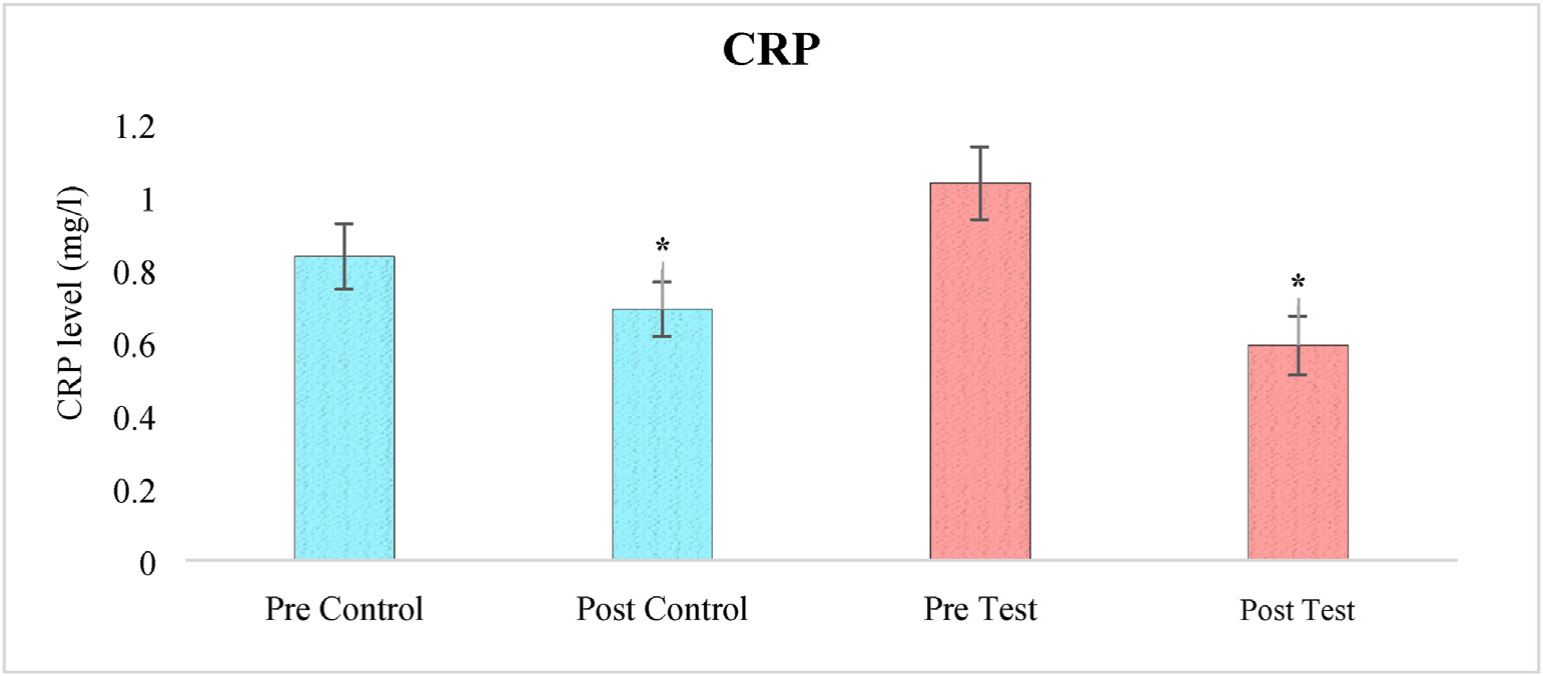
The effect of herbal tea on CRP levels. Values are presented as mean ± SD. Significant differences by Bonferroni test. ∗p < 0.05 as compared to respective pre-test group.

A one-way ANOVA analysis of CRP levels revealed a significant effect of treatment (F_3,236_ = 23.90, p < 0.05). At baseline, when the clinical study began, pair-wise comparisons using the Bonferroni test revealed a significant difference in CRP levels between the two groups (test and control). The level of CRP in post-test group was significantly lower than post control group (p < 0.05) indicated the anti-inflammatory response of herbal tea (Figure 4).

### 3.4 Effects on PHQ 9 scale

**Figure 5:**
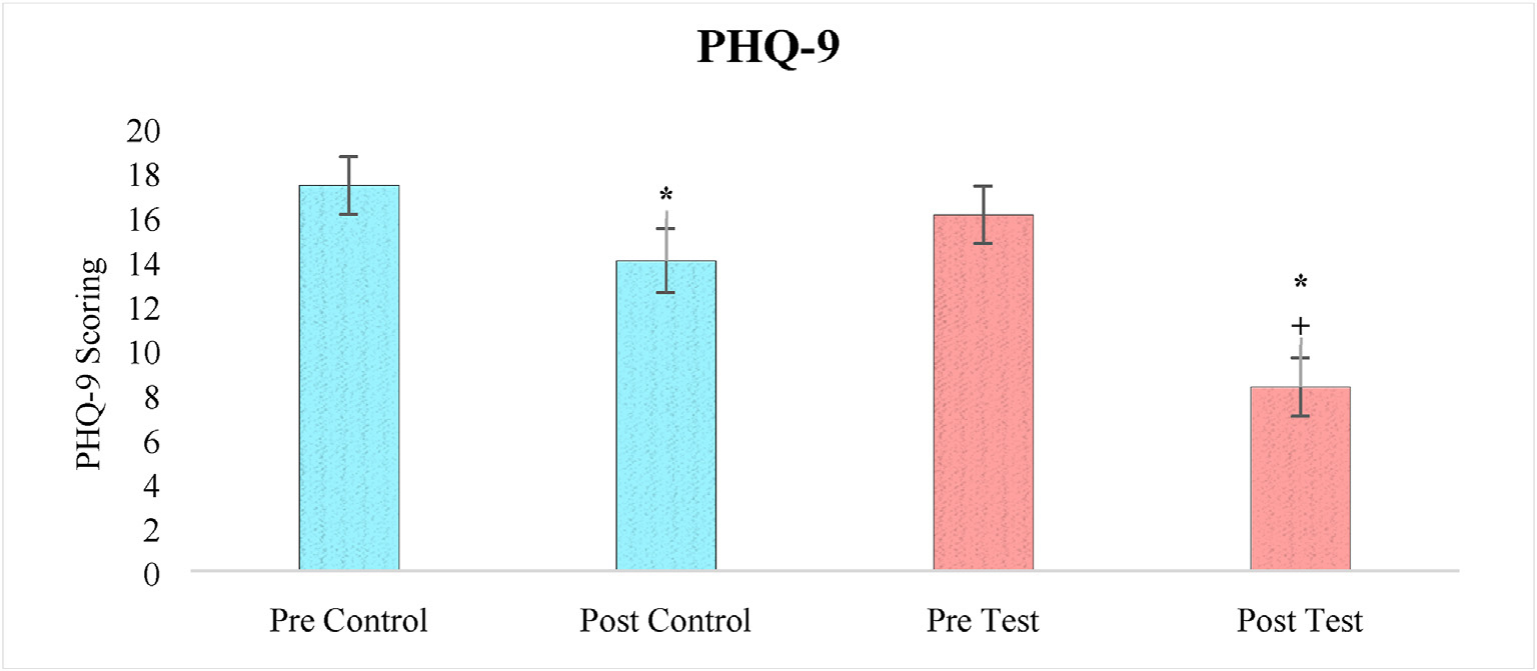
The effect of herbal tea on PHQ-9 score. Values are presented as mean ± SD. Significant differences by Bonferroni test. ∗p < 0.05 as compared to respective pre test group, +p < 0.05 as compared to post control group.

A one-way ANOVA analysis of PHQ-9 score revealed a significant effect of treatment (F_3,236_ = 33.11, p < 0.05). After one month of treatment, the severity of depressive behavior in both the control and test groups was significantly reduced (p < 0.05) compared to before treatment, according to a pair-wise comparison by Bonferroni test. However, when comparing the post test group to the post control group, it was discovered that the post test group showed a highly significant improvement (p < 0.05) (Figure 5).

## 4 Discussion

Depression is a common psychiatric condition characterized by mood disorders and caused by a loss of serotonergic neurotransmission (Sarris et al., 2011). However, the bioavailability of precursor amino acids is also a key concern, and their depletion can cause a range of neuronal alterations, including depression. Antidepressants are the first-line therapeutic option, as they restrict reuptake of neurotransmitters and thereby increase neurotransmitter levels in synapse. Recent research has found new molecular targets for depression and related metabolic disorders. As a result, novel remedies derived from natural and synthetic compounds are being tested to discover curative approach with cost-effective and long-term safety profile (Haenisch and Bönisch, 2011).

The present randomized clinical trial was designed to evaluate the effects of combined chamomile and saffron tea in depressed patients. The subjects were assigned to either control or test group. The participants of test groups were treated with herbal tea twice a day for one month along with their prescribed medications, while the control subjects were only relying on allopathic medications. Blood samples of all subjects were taken before and after the clinical trial. The outcomes reveals that the herbal tea showed the significant improvement to alleviate the depressive symptoms, which was recognized through PHQ-9 scale. Additionally, the blood samples were analyzed for TRP, BDNF and CRP levels to understand the therapeutic potential on biological system.

The results of present study demonstrate the effects of combined chamomile and saffron on depression parameters and it has been found that the herbal treatment of increase the availability of TRP in brain by decreasing the plasma concentration of TRP. The normal ingestion of protein containing <1 % TRP slightly increases the TRP level in plasma while greatly elevation of other LNNAs, thereby reduce the ratio of TRP/LNNAs and TRP influx to the brain. However, stress can also affect the availability of TRP in 5-HT producing cell since the activation of stress induced Kynurenic pathway which assist the connection between neuronal network and immune regulations (Chen and Miller, 2013). The elevated level of insulin increases the uptake of LNNA towards the skeletal muscle and thereby increase the TRP influx to the brain. Previous animal study has been found the strong potential effect of chamomile on insulin level, to interact with peroxisome proliferator-activated receptors in the liver, specifically PPARγ and improves insulin sensitivity throughout the body (Fernstrom, 1983). The action of safranal and crocin on 5-HT neuron and the elevation of -HT to produce antidepressant effect are well documented. Safranal and crocin is reported to act as non-competitive inhibitor of monoamine oxidase (MAO) A and B where it restricts the degradation of neurotransmitter. However, antidepressant effect is also attributed to the suppression of cortisol and serotonin transporter (SERT) level while raising tryptophan hydroxylase (TRH-2) level (Chen et al., 2008).

BDNF is neurotrophic factor critically important for neuronal growth and elicits significant role in excitation and inhibition of neurotransmitters by acting through tropomyosin receptor kinase-B (TrK-B) and support the survival of neuron by upregulating the synaptic proteins. The chronic treatment of herbal alternative is recently reported to elevate the level of BDNF in animal and human subjects (Zhu et al., 2018). Ghasemi et al., also found the neuroprotective effects of herbs including saffron on BDNF levels in brain (Ghasemi et al., 2015). Furthermore, it is reported that the macrophage migration inhibitory factor (MIF) regulates inflammatory responses and stimulates the expression of brain-derived neurotrophic factor (BDNF), which promotes neuron survival. Depression and other neuropsychological disorders may also be aggravated by malfunctioning of the MIF due to synthesis of free radicals and nitric oxide (NO) causing further harm of neuronal integrity and viability (Ruiz et al., 2022). The present study also found the anti-inflammatory response of herbal tea since the CRP level was seen to reduce in post test group than control subjects. CRP is nonspecific inflammatory marker, a cytokine which may induce obesity, diabetes, neurological and cardiovascular events. Topical aqueous chamomile extracts lowered tissue levels of (tumor necrosis factor) TNF- and (interleukin) IL-1, demonstrating anti-inflammatory effect, according to Curra et al. (2013). The plasma levels of MDD patients are found to represent higher CRP levels, higher neutrophil and monocyte counts, lower IL-10 levels, and a higher neutrophil to lymphocyte ratio (NLR) than controls subjects. Thus, possible associations between depression subtypes and its pathogenic characteristics with controls may be drawn through plasma levels of C-reactive protein (CRP), IL-6, IL-10, and leukocyte subpopulation in blood composition. In addition, lower IL-10 levels were linked to more severe depressed symptoms (Petralia et al., 2020a, 2020b).

The aqueous extract of chamomile inhibited the inflammatory impact generated by injection of prostaglandin E1 in albino rats, the inhibitory effects of chamomile aqueous extracts were comparable to those evoked by 10 mg/kg of the anti-allergenic drug oxatomide (Shipochliev et al., 1981). It has been summarized that the depression is common psychiatric illness and costly management, therefore innovative approaches are required to evaluate and establish to enhance the quality of lifestyle in larger scale with cost effective management, herbal products are best approaches to mitigate the symptoms. The present work demonstrated that the herbal alternatives of saffron and chamomile in the form of tea bags provided as the adjuvant therapy to the depressive patients with allopathic medicines provides strong antidepressant potential and improve the medicinal effects as well as treatment efficacy. Further studies are required to evaluate the effective of these herbs to establish the advanced therapeutic option for depression.

## 5 Conclusions

Saffron and chamomile have been used discretely as herbal medicine since ancient past and this study has shown that the co-administration of both herbs in adjuvant therapy led to better management of depression estimated through PHQ-9 questionnaire and related parameters such Tryptophan, BDNF, and CRP.

## 6 Limitations of the study

The major limitation of the study is the small sample size of patients. The study was done during COVID-19 so it highly restricted the approach of patients. The one of the major limitation is that the chamomile and saffron was given as herbal tea to the test group with their routine medications while the control were remained untreated with any herbal/placebo supplement, thus, future research could use placebo tea to compare the effects.

## Declarations

### Author contribution statement

Saara Ahmad: Conceived and designed the experiments; Performed the experiments; Analyzed and interpreted the data; Contributed reagents, materials, analysis tools or data; Wrote the paper.

Arfa Azhar; Prashant Tikmani; Hamna Rafique; Asra Khan: Performed the experiments; Analyzed and interpreted the data; Contributed reagents, materials, analysis tools or data; Wrote the paper.

Hanif Mesiya; Humera Saeed: Performed the experiments; Analyzed and interpreted the data; Wrote the paper.

### Funding statement

Dr Saara Ahmad Muddasir Khan was supported by 10.13039/501100004681Higher Education Commission, Pakistan [9447].

### Data availability statement

Data included in article/supp. material/referenced in article.

### Declaration of interest’s statement

The authors declare no conflict of interest.

### Additional information

The clinical trial described in this paper was registered at ClinicalTrials.gov. Identifier under the registration number NCT04935671.

Supplementary content related to this article has been published online at https://doi.org/10.1016/j.heliyon.2022.e10774.

## Appendix A Supplementary data

PHQ-9_English PHQ-9_English

PHQ 9 Urdu PHQ 9 Urdu
