# Supplementary material for: A randomized clinical trial to test efficacy of chamomile and saffron for neuroprotective and anti-inflammatory responses in depressive patients
Source: Heliyon. 2022 Sep 30;8(10):e10774. doi: 10.1016/j.heliyon.2022.e10774 (PMC9547202; doi:10.1016/j.heliyon.2022.e10774)
Supplement: PHQ 9 Urdu [file mmc2.pdf]

Office use only | | **PHQ-9**

نام ______________________ تاریخ ______________

گزشتہ 2 ہفتوں کے دوران، آپ کو درجِ ذیل مسائل کی وجہ سے کتنی مرتبہ مشکل پیش آئی ہے؟

PHQ-9	خانے میں اپنے جواب کی نشاندہی کرنے کے لئے " ✓ " استعمال کیجئے	بالکل نہیں	کئی دن	آدھے دن سے زیادہ	تقریباًروزانہ
1	کچھ بھی کرنے میں بہت کم دلچسپی یا خوشی ہوتی ہے۔	0	1	2	3
2	پژمردہ، افسردہ یا ناامید ہونا۔	0	1	2	3
3	سونے یا سوئے رہنے میں مشکل ہونا، یا بہت زیادہ سونا۔	0	1	2	3
4	تھکان محسوس کرنا یا بہت کم توانائی محسوس کرنا۔	0	1	2	3
5	بھوک میں کمی یا بہت زیادہ کھانا۔	0	1	2	3
6	اپنے متعلق برا محسوس کرنا ۔ یا یہ کہ آپ ناکارہ ہیں یا آپ نے خود کو یا اپنے خاندان کو مایوس کیا ہے۔	0	1	2	3
7	کسی کام میں توجہ مرکوز کرنے میں مشکل ہونا، جیسے کہ اخبار پڑھنا یا ٹی وی دیکھنا۔	0	1	2	3
8	اتنا آستہ حرکت یا بات کرنا جسے دوسرے لوگ محسوس کر سکتے تھے؟ یا اس کا الٹ ۔ اتنی بے چینی محسوس کرنا کہ آپ معمول سے زیادہ حرکت کریں۔	0	1	2	3
9	اس قسم کے خیالات آنا کہ آپ کا مر جانا ہی بہتر ہے یا خود کو کسی طریقے سے تکلیف پہنچانے کے بارے میں سوچنا۔	0	1	2	3
